# FuzzyEn Compared to SampEn for Evaluation of Dynamic Complexity

**DOI:** 10.3390/e28080841

**Published:** 2026-07-28

**Authors:** Xingyu Liu, Mingyuan Su, Wenpo Yao, Yaru Dong

**Affiliations:** School of Chemistry and Life Sciences, Nanjing University of Posts and Telecommunications, Nanjing 210023, China

**Keywords:** dynamic complexity, fuzzy entropy, sample entropy, depressed EEG

## Abstract

Fuzzy entropy (FuzzyEn) theoretically outperforms sample entropy (SampEn) in quantifying dynamic complexity; however, its anti-noise robustness remains insufficiently validated. This study compares SampEn and FuzzyEn using model simulations and real-world depression electroencephalogram (EEG) signals. The two entropy metrics are first compared using chaotic time series generated from the logistic and two-dimensional Henon maps, with additive white Gaussian noise (SNRs ranging from 1 to 10 dB). Surrogate data analysis reveals that SampEn maintains stronger nonlinear detection performance (lower than the 2.5th percentiles of surrogate data) under noisy conditions. EEG data from 46 depressed patients and 75 healthy subjects are employed to evaluate SampEn and FuzzyEn, with statistical differences corrected by the Bonferroni method. Depressed patients showed significantly increased EEG complexity in the occipital lobe (PO7, PO5, PO3 channels) under visual stimulation (*p* < 0.01). SampEn outperforms FuzzyEn in alpha-band feature detection, especially the low-alpha sub-band (8–10 Hz, *p* < 0.001). In summary, this study verifies that SampEn may be more suitable than FuzzyEn under noise conditions, thus providing valuable insights for real-world signal analysis and methodological guidance for developing EEG biomarkers of depression.

## 1. Introduction

Entropy metrics, rooted in information theory, are widely used to quantify dynamic complexity [1,2]. Among them, sample entropy (SampEn) is a predominant tool due to its quantification performance [3,4]. As an optimized variant proposed by Chen et al. [5,6], fuzzy entropy (FuzzyEn) employs fuzzy boundaries rather than step thresholds. While fuzzy similarity theoretically enhances measurement precision, FuzzyEn exhibits greater vulnerability to interferences. This inherent drawback lacks systematic verification in prior research, restricting holistic insights into their merits for practical signal analysis.

Both SampEn and FuzzyEn serve as concrete implementations of conditional entropy. SampEn was developed to overcome the drawbacks of approximate entropy (ApEn) [3,4,7], featuring reliable performance for short time series and low sensitivity to parameter variation [4,8]. As proposed by Chen et al. [5,6], FuzzyEn introduces fuzzy set theory [9,10] to quantify vector similarity during probability estimation. The fuzzy similarity metric theoretically enables FuzzyEn to achieve higher quantification precision, a property that has been corroborated by subsequent research. Simons et al. [11] reported that FuzzyEn outperformed ApEn and SampEn in Alzheimer’s disease diagnosis based on electroencephalogram (EEG). With properly configured input parameters, FuzzyEn can offer deeper insights into the neural dysfunctions underlying Alzheimer’s disease. According to Chen et al. [12], FuzzyEn is more effective in capturing the dynamical complexity of depressive EEG signals and yields statistically significant intergroup differences within the theta and alpha frequency bands. Considering the scale factors inherent in complex systems, Cao and Lin [13] and Wang et al. [14] adopted multiscale strategies to enhance the reliability of real-time EEG analysis. In summary, existing research on FuzzyEn consistently demonstrates its superior accuracy in quantifying dynamical complexity.

Compared with precise fuzzy computation, conventional coarse-grained strategies yield stronger anti-noise robustness and greater computational convenience. Ordinal patterns represent temporal structure, and related entropy metrics—including static permutation entropy and dynamic sort entropy [15]—as well as time irreversibility [16] exhibit strong anti-noise capability and high computational efficiency. In contrast, fuzzy permutation methods [17] suffer from higher implementation complexity and greater susceptibility to interference. Compared to symbolic analysis and step threshold filters [18], the fuzzy distribution of equal states (fDES) shares the same limitations [19]. Existing research on fuzzy theory, ranging from fundamental theoretical investigations to applied numerical analysis works, consistently confirms that fuzzy methods are characterized by high implementation complexity [10,19,20,21,22]. Despite the loss of some detailed information when performing the low-passing selection of vector distances, the coarse-graining SampEn features simple implementation and should have an anti-interference advantage. Nevertheless, few existing works systematically compare FuzzyEn and SampEn in terms of noise robustness and the reliability of nonlinear detection.

To address these issues, this study systematically compares SampEn and FuzzyEn in both simulation experiments and real-world EEG analysis. Synthetic chaotic signals are generated with noise of varying signal-to-noise ratios, and surrogate data tests are adopted to evaluate SampEn and FuzzyEn. Depressive EEG data and their frequency band components [23] are derived to characterize dynamic complexity detections between the two entropy measures comparatively. Fundamental distinctions in the noise resilience and nonlinear characterization capabilities of the two entropy algorithms are compared. Overall, our work elaborates comparative theoretical and numerical properties between SampEn and FuzzyEn and provides methodological support for the development of robust EEG biomarkers for depression.

## 2. Sample Entropy and Fuzzy Entropy

### 2.1. Conditional Entropy

The concept of information entropy was first introduced by Claude Shannon in 1948 in his seminal work *A Mathematical Theory of Communication*, where Shannon entropy was defined to quantify the uncertainty and information content of a process. Entropy is a concept in information theory that is analogous to the definition in statistical thermodynamics. Given process $X\left(t\right)=\left\{x_{1},x_{2},\ldots ,x_{t},\ldots ,x_{L}\right\}$ with discrete variables whose probability is p(x), its Shannon entropy is defined as the average information measured by the negative logarithm of p(x) as in Equation (1)

(1) $$\begin{array}{c} H(X)=-\sum p(x)logp(x) \end{array}$$

Building on Shannon’s framework, Kolmogorov and Sinai measured the rate of information generation in chaotic dynamical systems, laying the theoretical foundation for conditional entropy [1,2]. For a dynamic process, the present state is partially determined by its past states, and it contains information that cannot be inferred from the past history. In information theory, the present state could be defined as $X_{n}$ and the past as $X_{n}^{-}=\left\{X_{1},X_{2},\ldots ,X_{n-2},X_{n-1}\right\}$; the average rate of creation of new information in the present state is conditional entropy C(X). The conditional entropy of $X_{n}$ and $X_{n}^{-}$ is the quantity of present information that cannot be provided by the given past of dynamical systems. It is given by Equation (2).

(2) $$\begin{array}{ccc} C(X) & = & H\left(X_{n}|X_{n}^{-}\right)=H\left(X_{n},X_{n}^{-}\right)-H\left(X_{n}^{-}\right)\\ & = & -E\left[logp(x_{n}|x_{1},\ldots ,x_{n-2},x_{n-1})\right] \end{array}$$

The notion of conditional entropy for dynamic systems is an interesting entropy transplanted from Shannon entropy and is widely adopted by quantitative parameters of dynamic complexity, such as ApEn, SampEn and FuzzyEn [3,4,5,6,7].

### 2.2. Sample Entropy

SampEn is an implementation of conditional entropy and an improved algorithm of ApEn [3,4]. SampEn has been improved in terms of two aspects: it excludes self-matching, j≠i; it does not use a template-wise approach and only matches dimension m+1. Given $X\left(t\right)=\left\{x_{1},x_{2},\ldots ,x_{t},\ldots ,x_{L}\right\}$, multi-dimension vectors are constructed as

(3) $$\begin{array}{c} X_{\tau }^{m}\left(t\right)=\left\{x_{\tau }^{m}\left(1\right),x_{\tau }^{m}\left(2\right),\ldots ,x_{\tau }^{m}\left(L-\left(m-1\right)\tau \right)\right\} \end{array}$$

with dimension *m* and delay τ, where $x_{\tau }^{m}\left(t\right)=\left\{x\left(t\right),x\left(t+\tau \right),\ldots ,x\left(t+\left(m-1\right)\tau \right)\right\}$. The Chebyshev distance of $x_{\tau }^{m}\left(i\right)$ and $x_{\tau }^{m}\left(j\right)$, i.e., the maximum norm $d_{\left(i,j\right)}$, is computed as

(4) $$\begin{array}{c} d_{i,j}=∥x_{\tau }^{m}\left(i\right)-x_{\tau }^{m}\left(j\right)∥=\underset{k\epsilon (0,m-1)}{max}\left|x\left(i+k\right)-x\left(j+k\right)\right|. \end{array}$$

A threshold $r=k*\sqrt{\sum {\left(x\left(t\right)-\bar{x}\right)}^{2}/\left(L-1\right)}$, also called tolerance in the other literature, is set, where $\bar{x}$ is the mean of series, and *k* is an adjustable parameter. To compare the similarity of vectors for probability estimation, the Heaviside kernel is employed to compare their distance and threshold r, i.e., the low-pass filtering of vector distances, as

(5) $$\begin{array}{c} K\left(d_{i,j},r\right)=\Theta \left(r-d_{i,j}\right)=\left\{\begin{array}{c} 0,\;d_{i,j}>r\\ 1,\;d_{i,j}\leq r \end{array}\right. \end{array}$$

$B_{r}^{m}\left(i\right)=\frac{1}{N-m-1}num\left\{k=1\right\}$ where num{k=1} is defined as the number of vectors $x_{\tau }^{m}\left(j\right)$ within the tolerance of $x_{\tau }^{m}\left(i\right)$. To reduce the bias caused by including self-matches, j≠i is restrained in $B_{r}^{m}\left(i\right)$. The probability associated with dimension *m* is $B^{m}\left(r\right)=\frac{1}{N-m-1}\sum_{i=1}^{N-m}B_{r}^{m}\left(i\right)$.

Corresponding to $B^{m}\left(r\right)$, the dimension is increased to m+1, and the updated probability $B^{m+1}\left(r\right)$ is measured. Multi-dimension vectors $X_{\tau }^{m+1}\left(t\right)$ are further constructed, and their Chebyshev distances are filtered by the tolerance *r*. The probability associated with dimension m+1 is denoted as B(m+1)(r). The SampEn of X(t) is defined as

(6) $$\begin{array}{c} SampEn\left(m,r,\tau \right)=-(ln\left(B^{m+1}\left(r\right)\right)-ln\left(B^{m}\left(r\right)\right)) \end{array}$$

### 2.3. Fuzzy Entropy

In the real world, classification sometimes is fuzzy but not crisp, as classes may not have precise criteria. Fuzzy sets dealing with the ambiguity of classes come with a continuum of grades of membership, with no abrupt change in the conventional crisp membership function [10]. Chen et al. [5,6] proposed FuzzyEn by adopting a family of exponential functions without changes in similarity and convexity, thereby maximizing self-similarity. SampEn and FuzzyEn are different in terms of transforming vector distance in probability estimation. SampEn involves counting similar patterns within a specified tolerance level, while FuzzyEn requires defining membership functions for fuzzy sets and computing entropy based on these memberships.

Multi-dimension vectors $X_{\tau }^{m}\left(t\right)$ are constructed with dimension *m* and delay τ, and the Chebyshev distance $d_{i,j}$ of $x_{\tau }^{m}\left(i\right)$ and $x_{\tau }^{m}\left(j\right)$ is calculated. Unlike the binary Heaviside function, FuzzyEn uses a continuous exponential function to measure the similarity between vectors. In FuzzyEn, the similarity between vectors is measured using an exponential function, as shown in Equation (7).

(7) $$\begin{array}{c} K\left(d_{i,j},r\right)=exp\left(-{\left(d_{i,j}/r\right)}^{2}\right) \end{array}$$

**Figure 1:**
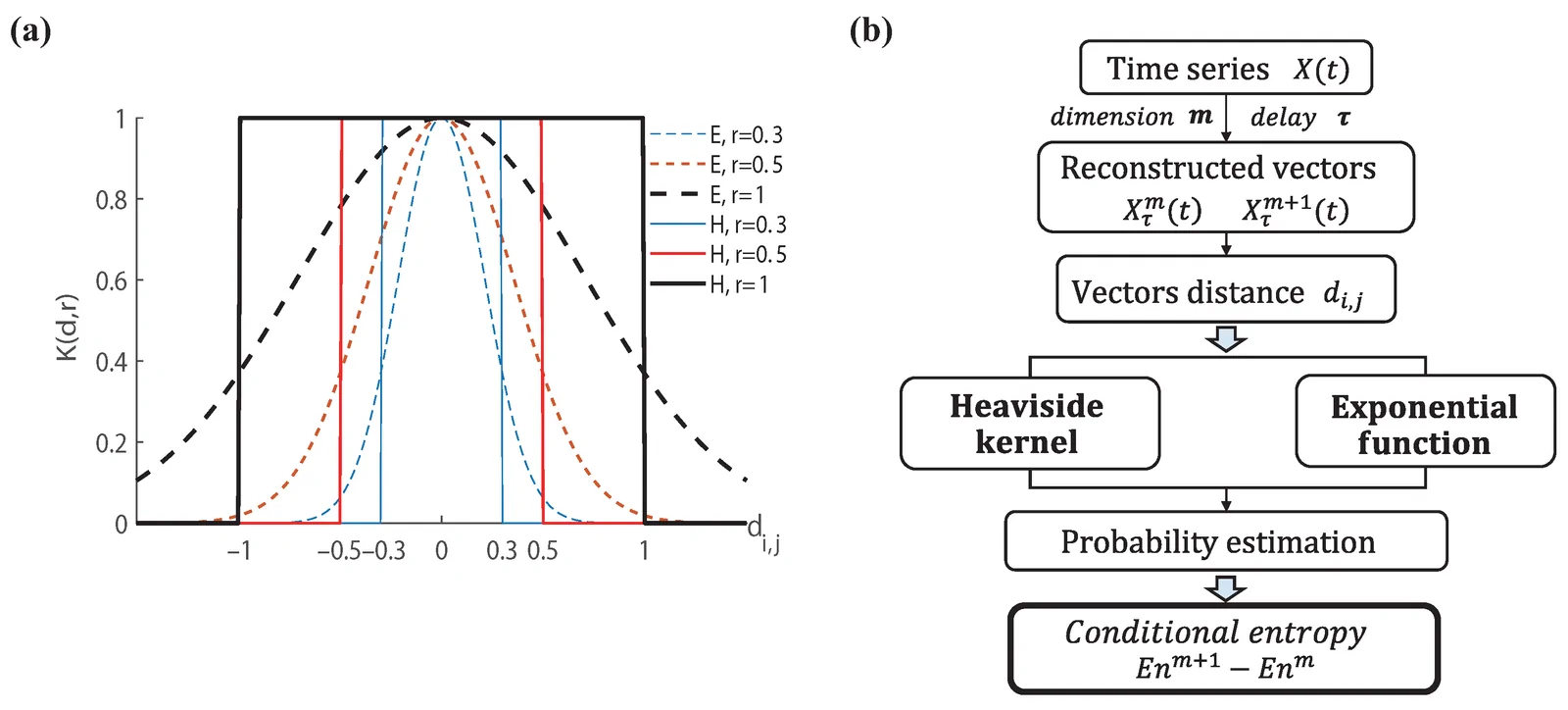
Comparison of SampEn and FuzzyEn. (**a**) Heaviside (denoted as ‘H’) and exponential (denoted as ‘E’) functions for vectors distance; (**b**) flowchart of SampEn and FuzzyEn.

As shown in Figure 1, SampEn and FuzzyEn differ in terms of handling vector distances for probability estimation. The Heaviside kernel function uses a coarse-grained, low-pass filtering of vector distances, assigning 1 to all distances within the predefined threshold *r*. In contrast, negative exponential filtering (for FuzzyEn) gives a maximum contribution of 1 to probability estimation when two vectors are identical (distance = 0), with this contribution decreasing as vector distance increases.

We define $F_{r}^{m}\left(i\right)=\frac{1}{N-m-1}\sum_{j=1}^{N-m}K\left(d_{i,j},r\right)$ and $D^{m}\left(r\right)=\frac{1}{N-m-1}\sum_{i=1}^{N-m}F_{r}^{m}\left(i\right)$. Then, we increase dimension to m+1 and calculate $D^{m+1}\left(r\right)$; FuzzyEn is measured as shown in Equation (8).

(8) $$\begin{array}{c} FuzzyEn\left(m,r,\tau \right)=-(ln\left(D^{m+1}\left(r\right)\right)-ln\left(D^{m}\left(r\right)\right)) \end{array}$$

It can be observed that the main difference between SampEn and FuzzyEn lies in the conversion of vector distances in probability estimation, which determines the accuracy of entropy calculation. Theoretically, FuzzyEn is more accurate in measuring the differences between vectors and therefore measures the probability distribution of the sequence with greater accuracy [5,6,11,13]. However, SampEn only filters vector distances with a hard threshold and counts vectors falling within the cutoff when estimating probabilities. In contrast, FuzzyEn incorporates all pairwise vector distances and computes membership degrees via negative exponential functions. For this reason, SampEn is easier to implement on practical software and hardware platforms.

## 3. Results

In this section, we first generate model series to evaluate SampEn and FuzzyEn and then compare them to analyze real-world depressed EEG signals.

### 3.1. Model Tests in Chaotic Sequences

We first generated chaotic sequences via the logistic map, mathematically expressed as $x_{t+1}=r\cdot x_{t}\left(1-x_{t}\right)$ with *r* = 4, and two-dimensional Henon map $x_{t+1}=1-\alpha \cdot x_{t}^{2}+y_{t}$, $y_{t+1}=\beta \cdot x_{t}$ with α = 1.4, β = 0.3. The initial values are $x_{1}$ = $y_{1}$ = 0.01, and the $x_{t}$ of the Henon series was utilized. Both chaotic sequences were generated for 10,000 sample points. Additive white Gaussian noise (AWGN) was introduced to two sets of chaotic series as $x_{t}^{\prime }=x_{t}+\xi_{t}$, where $\xi_{t}$ denotes a zero-mean, unit-variance Gaussian random variable.

According to surrogate data theory, if the discriminating index (here, entropy values) of the original process is less than the 2.5th percentile or greater than the 97.5th percentile of the surrogate dataset, the null hypothesis (i.e., the series is linear and stochastic) is false and should be rejected. Surrogate data methods are commonly adopted to validate the effectiveness of proposed nonlinear approaches using signals with well-characterized properties or to detect nonlinear characteristics within unknown signals, with about 100 surrogate datasets typically constructed to guarantee statistically reliable validation [24]. In this work, we employed chaotic Logistic and Henon series, while both FuzzyEn and SampEn serve as metrics for quantifying nonlinear complexity. A total of 500 sets of surrogate data, preserving the autocorrelation structure and power spectral density of the original series, were constructed using the model-free improved amplitude-adjusted Fourier transform (iAAFT) method [25,26]. Accordingly, we innovatively introduced additive noise and surrogate data theory to systematically compare the noise robustness of these two entropy measures.

**Figure 2:**
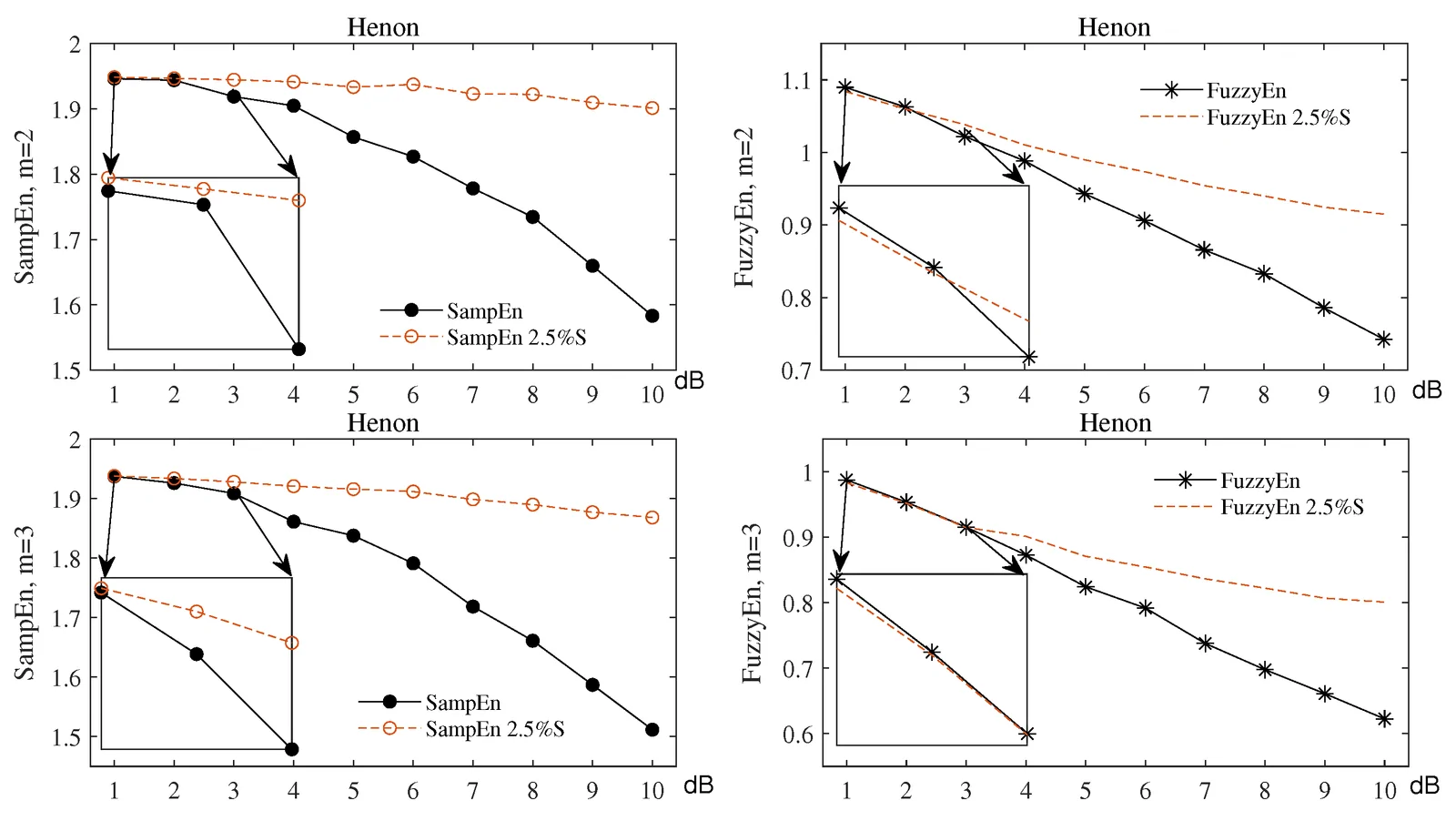
The FuzzyEn and SampEn of the Henon series and its surrogate data under different noise levels, with arrows pointing to magnified graphical results for the SNR ranging from 1 dB to 3 dB.

In line with established protocols in entropy analysis, the parameters were set as follows: embedding dimension *m* = 2 and 3, τ = 1 and *r* = 0.25 [3,4,5,6,27]. The FuzzyEn and SampEn of the noise-free logistic and Henon series were both lower than the 2.5th percentiles of the surrogate data. The surrogate data generated by iAAFT exhibit stochastic and unpredictable characteristics with high dynamical complexity. In contrast, the logistic and Henon series are deterministic chaotic nonlinear signals. For this reason, their corresponding SampEn and FuzzyEn values are lower than the 2.5th percentiles calculated from the surrogate ensemble. Subsequently, AWGN was added to the logistic and Henon series at signal-to-noise ratios (SNRs) ranging from 1 to 10 dB, and surrogate data were reconstructed following the iAAFT procedure. FuzzyEn/SampEn values for *m* = 2 and 3 (denoted as *m*2 and *m*3) were computed, the results of logistic are summarized in Table 1, and those of Henon are shown in Figure 2.

Table 1 and Figure 2 demonstrate that SampEn exhibits consistently superior anti-noise robustness compared with FuzzyEn. Specifically, the SampEn values of the noisy logistic series fall below the 2.5th percentiles of the corresponding surrogate data at an SNR ≥ 5 dB for *m* = 2. By contrast, FuzzyEn fails to satisfy this detection criterion until reaching an SNR ≥ 9 dB. When *m* = 3, more data points are involved in phase space reconstruction and probability distribution estimation, which makes entropy metrics intrinsically more susceptible to noise interference. SampEn achieves valid nonlinearity detection at an SNR ≥ 8 dB, whereas FuzzyEn requires a minimum SNR ≥ 10 dB.

For Henon series, SampEn consistently produces entropy values smaller than the 2.5th percentiles of surrogate data across both *m* = 2 and 3 over all tested noise levels. As for FuzzyEn, valid nonlinear identification is only attained when SNR > 2 dB for *m* = 2, and the required signal quality further rises to SNR > 3 dB at *m* = 2. Such statistical outcomes reject the null hypothesis that the time series originates from linear stochastic processes, demonstrating that SampEn retains robust capability for nonlinear feature identification under moderate noise corruption.

Collectively, these results confirm that FuzzyEn is more vulnerable to the noise-induced distortion of nonlinear feature extraction than SampEn, making SampEn a more robust metric for noisy chaotic time series analysis.

### 3.2. Dynamic Complexity of Depressed EEGs

Depression (also known as depressive disorder) is a common mental disorder that can occur in all age groups, cause difficulties in all aspects of life, and can even lead to suicide [28]. It occurs due to the interaction of social, psychological, and biological factors. The human brain is a collection of a large number of neurons interacting with each other and subjected to physiological conditions. Considering the high complexity of the brain, entropy measures are gaining popularity in EEG nonlinear dynamic analysis [29,30,31,32]. Depressive EEG recordings are collected from the public PRED+CT database, based on which a comparative analysis of FuzzyEn and SampEn for dynamic complexity detection is conducted.

Based on the mass survey scores of the Beck Depression Inventory (BDI) [33], 46 depressed patients with a stable high BDI score (≥13) (aged 18 to 24, 18.74 ± 1.14 years, including 34 females) and 75 control participants with a stable low BDI score (<7) (aged 18 to 23, 18.97 ± 1.22 years, including 40 females) were recruited from introductory psychology classes. During the experiment, participants were subject to two distinct conditions, specifically the ‘Rest’ and ‘Reinforcement Learning’ conditions. During the ‘Rest’ experiment, participants were guided to open and close their eyes following instructions. During the ‘Reinforcement Learning’ experiment, participants performed a learning task: three pairs of a Japanese Hiragana character associated with different feedback were presented during training, and then stimulus pairs were presented eight times for testing. Brain electric activity was collected in the training phase about responses to feedback during learning. Surface electrodes were placed according to the international 10–20 system, and 60 total channels contributed to EEG recordings with a band-pass filter of 0.5–100 Hz and a 500 Hz sampling rate. Given the requirements of entropy estimation to time series length, we uniformly adopted 30 s EEG segments containing 15,000 sampling points. Artifacts from eye movements were removed using ICA. Data were then referenced to averaged mastoids. Note that the ‘Rest’ control dataset omits subjects no. 571 and no. 572, and the ‘Reinforcement Learning’ depressive EEG dataset excludes participants no. 599 and no. 600 in the public database. Detailed information about the depression EEGs can be found in Ref. [23].

To compare the statistical differences in EEG signal entropy values between the ‘Rest’ and ‘Reinforcement Learning’ states in the same participants, the nonparametric Wilcoxon signed-rank test was adopted. Separately, the Mann–Whitney U test was used to analyze the statistical differences in EEG entropy values between depressed patients and healthy control subjects. Furthermore, considering the interaction effects across different participants and experimental states, a permutational mixed analysis of variance was also performed to test the statistical disparities of entropy values for all EEG signals. To eliminate false positive errors in the case of multiple comparison (i.e., 60 channels of EEG signal acquisition) [34,35,36], the Bonferroni correction for statistical differences is used, in which p-values are multiplied by 60 in the following analysis.

**Figure 3:**
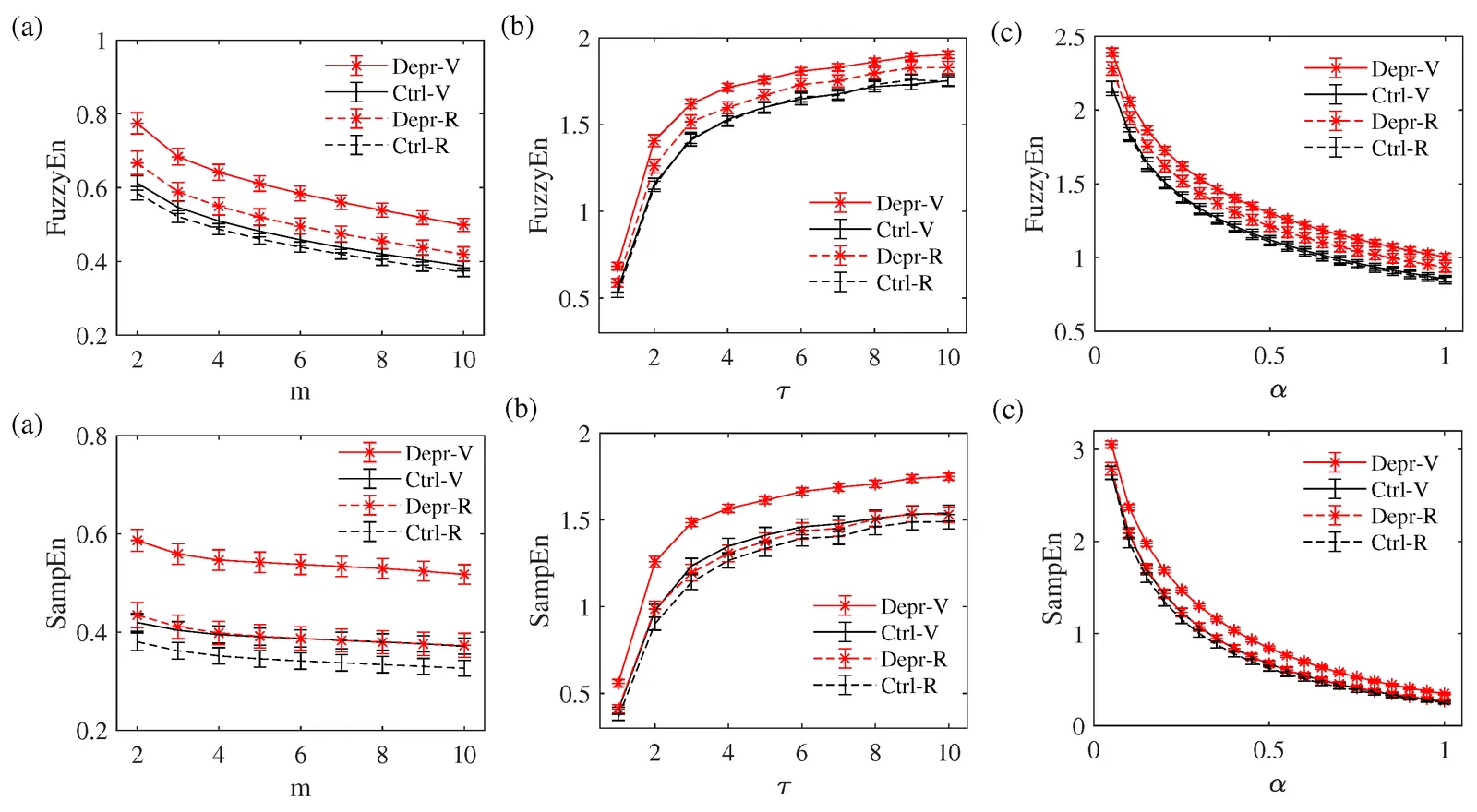
Determination of parameter of FuzzyEn and SampEn (mean ± standard error) based on PO5. (**a**) Selection of dimension when τ = 1, *r* = 0.25; (**b**) selection of delay based on *m* = 3, *r* = 0.25; (**c**) control parameter of tolerance when *m* = 3, τ = 3. ‘Depr-R’ and ‘Depr-V’ denote depressed EEGs in rest and visual-stimulated learning states, and ‘Ctrl-R’ and ‘Ctrl-V’ denote those of healthy control.

Consistent with the previous model tests, the initial parameters of FuzzyEn and SampEn were set to *m* = 2, τ = 1 and *r* = 0.25 [3,4,5,6,27]. We determined the optimal control parameters for SampEn and FuzzyEn via a trial-and-error search scheme covering all 60 whole-brain channels. Among these channels, the PO7, PO5, and PO3 channels displayed significant discriminative capacity (*p* < 0.01), with the PO5 channel yielding the most optimal discriminative performance (Bonferroni-corrected *p* = 0.005). Using the PO5 channel for example, the FuzzyEn and SampEn of PO5 EEGs with varying parameters are shown in Figure 3.

Given the importance of dimension in vector reconstruction and entropy calculation, we first detect optimal dimension from 2 to 10 with τ = 1 and *r* = 0.25. The increase in dimension increases the amount of space vector elements, which in turn increases the computational complexity of vector distance. The FuzzyEn values of EEG signals exhibit a relatively sharp decrease when *m* = 3, after which the variation becomes gentle. SampEn became convergent when the dimension is 3 or larger. Considering the computational consumption and stability of the results, we selected the dimension 3 as the optimal choice.

A delay factor that is too large (equivalent to reduction in sampling frequency to f/τ) could cause a loss of information correlation (irrelevance). Given the sampling frequency of 500 Hz and a band-pass filter of 0.5–100 Hz, the delay factor should be no larger than 5. According to Figure 3, the FuzzyEn and SampEn of EEGs had a large up-trend change when the delay factor was less than 3 and became convergent when the delay was 3 or larger. Therefore, we selected 3 as the optimal delay factor.

Finally, *r* is set from 0.05 to 1, with a step of 0.05 based on *m* = 3 and τ = 3. When tolerance was between 0.1 and 0.3, statistical discriminations among dynamic complexities of the four groups of EEGs showed no significant changes. Larger tolerances reduce the accuracy of determination of equal vectors as well as probability estimation; therefore, we selected the control parameter of tolerance to be 0.15.

The FuzzyEn and SampEn of EEGs at other channels had consistent changes with control parameters, and statistical differences were not significantly affected. Insensitivity to parameter selection is critical in signal processing, especially for the analysis of signals from complex or unknown systems.

Based on the selected parameters, i.e., *m* = 3, τ = 3 and *r* = 0.15, we conducted a comparative analysis of SampEn and FuzzyEn for the depressed EEG signal dynamic complexity analysis.

**Figure 4:**
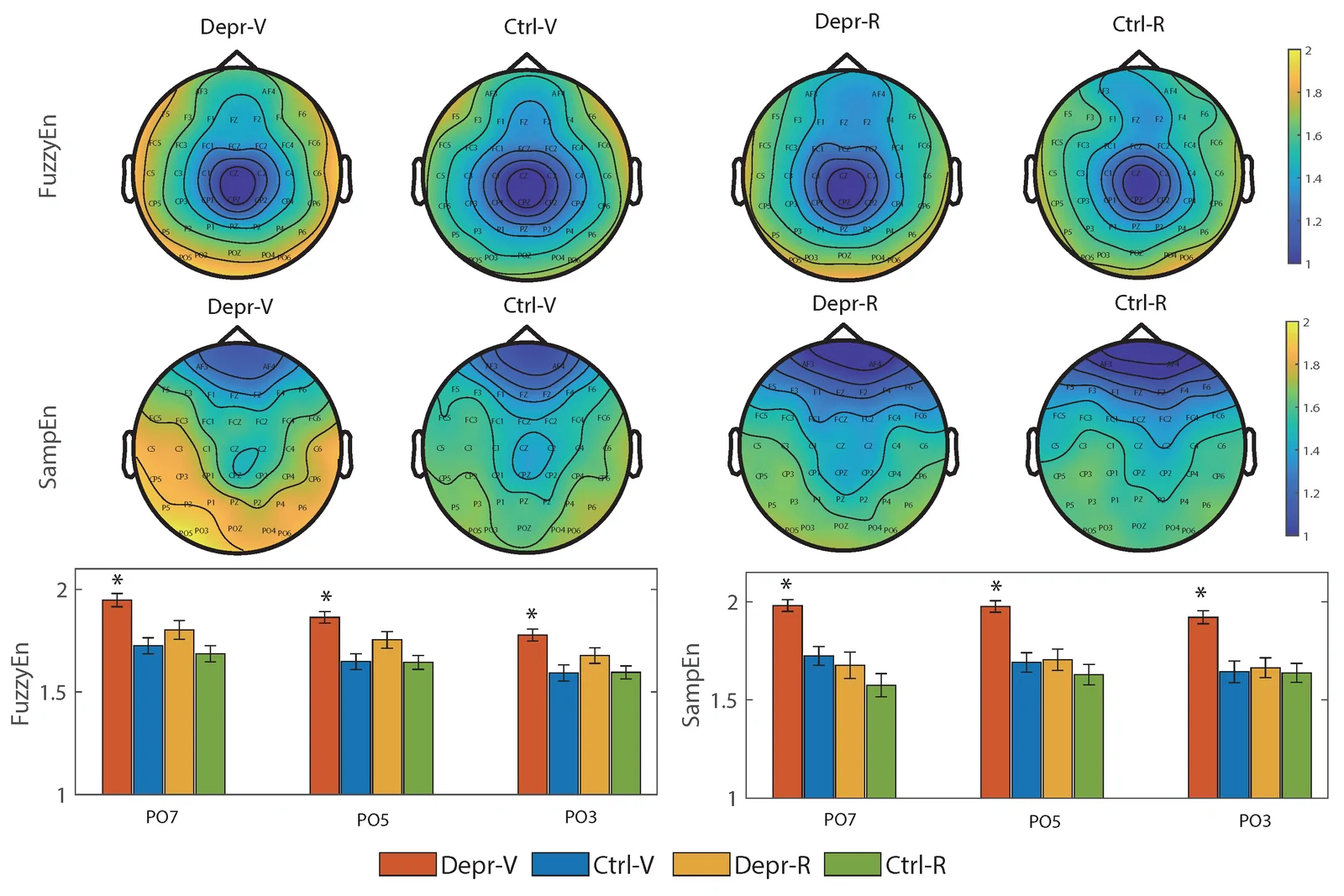
SampEn and FuzzyEn of depressed EEGs with *m* = 3, τ = 3 and *r* = 0.15. Circled PO7, PO5 and PO3 in left occipital lobes are shown due to optimal discrimination. ‘Depr-R’ and ‘Depr-V’ denote depressed EEGs in rest and visual-stimulated learning states, and ‘Ctrl-R’ and ‘Ctrl-V’ denote those of healthy control. ‘*’ represents case in which entropy of EEGs is significantly different from that of others (Bonferroni-corrected *p* < 0.01).

In Figure 4, depressed EEGs under visual stimulation have significantly higher complexity than those in the rest state and than those recorded from healthy controls, especially in PO3, PO5 and PO7 electrodes. Specifically, FuzzyEn outperformed at PO3, while SampEn outperformed at PO5 and PO7. Further statistical analysis focusing on the PO3, PO5, and PO7 electrode key sites for monitoring occipital EEG activity related to depression revealed no differences in the recognition accuracy of depressive EEGs.

The results demonstrated a high degree of consistency between FuzzyEn and SampEn. During the resting state, no statistically significant difference was detected in the dynamic complexity of depressed and healthy control EEG signals. In contrast, upon exposure to visual stimulation, the dynamic complexity of depressed EEGs increased significantly. More importantly, the dynamic complexity of depressed EEGs was significantly greater than that of the healthy control group, and this difference was particularly pronounced in the left occipital lobe. Occipital EEG activity was stimulated in the visual experiment [37,38], during which the depressed EEGs were more complex. In the framework of nonlinear dynamics, elevated FuzzyEn and SampEn values are indicative of increased dynamic complexity in neural signals, reflecting more irregular patterns of neural activity. This reveals that in response to the visual stimulation of Japanese characters, depressed patients exhibit a stronger neural response in the occipital visual area. Such enhanced responsiveness may stem from alterations in visual processing mechanisms associated with depression, highlighting potential neurophysiological differences in how this population perceives and processes complex visual–linguistic stimuli.

### 3.3. Frequency Band Complexity Alterations in Depressed EEGs

We further extracted frequency components corresponding to delta (0.5–4 Hz), theta (4–8 Hz), alpha (8–13 Hz), beta (13–30 Hz) and gamma (>30 Hz) from the EEG signals. We then comparatively applied SampEn and FuzzyEn to analyze EEG signals in the frequency bands for patients with depression. SampEn and FuzzyEn exhibited low sensitivity to parameter variations. Thus, we adopted the optimally tested parameter set identified in our preliminary experiments, specifically *m* = 3, τ = 3 and *r* = 0.15.

Across all 60 recording channels, inconsistent disparities in dynamic complexity were observed between the delta (0.5–4 Hz) and theta (4–8 Hz) EEG signals. Delta rhythms are classically associated with deep non-rapid eye movement sleep and exhibit maximal amplitude over frontal–central scalp regions. Our findings revealed that delta band oscillations were not associated with depression. The absence of discriminative features in delta band EEGs indicates that the delta band is insufficient in characterizing the neurophysiological alterations in depression.

**Figure 5:**
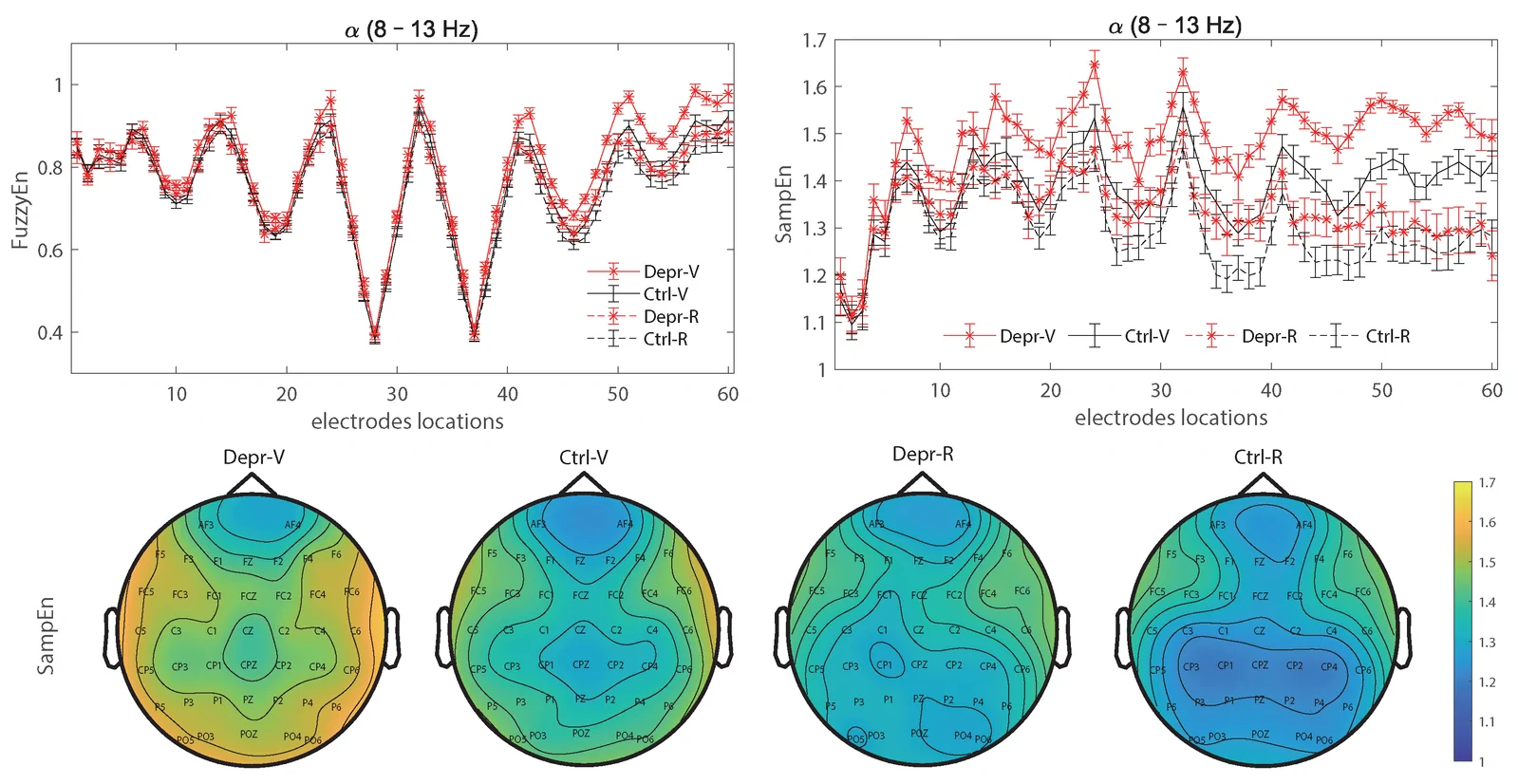
SampEn and FuzzyEn of depressed EEGs (mean ± standard error) in α (8–13 Hz) bands. SampEn brain topography maps are presented below. Electrode locations 1–60 in x-coordinate represent FP1, FPZ, FP2, AF3, AF4, F7, F5, F3, F1, FZ, F2, F4, F6, F8, FT7, FC5, FC3, FC1, FCZ, FC2, FC4, FC6, FT8, T7, C5, C3, C1, CZ, C2, C4, C6, T8, TP7, CP5, CP3, CP1, CPZ, CP2, CP4, CP6, TP8, P7, P5, P3, P1, PZ, P2, P4, P6, P8, PO7, PO5, PO3, POZ, PO4, PO6, PO8, O1, OZ, and O2 respectively.

In the alpha band (8–13 Hz) EEG signals, both entropy metrics demonstrated significant discriminative capacity for distinguishing EEG complexity in the occipital region between depressed patients and healthy controls. SampEn outperformed FuzzyEn significantly in identifying more discriminative electrodes, as illustrated in Figure 5. This indicates that alpha band oscillations are vital in depression-related neurophysiological alterations, particularly in the context of visual processing tasks such as Japanese language stimuli. This strong association underscores the alpha band’s potential as a key target for a further entropy-based analysis of dynamic complexity in depression research. Our findings are consistent with previous research noting that alpha-based features provide high accuracy in classification [39,40].

In the beta (13–30 Hz) and gamma (>30 Hz) bands, neither SampEn nor FuzzyEn could distinguish the complexity characteristics of EEG signals between the study groups. This finding suggests that the neurophysiological alterations underlying depression are easily studied in lower oscillatory rhythms, especially during visual tasks.

**Figure 6:**
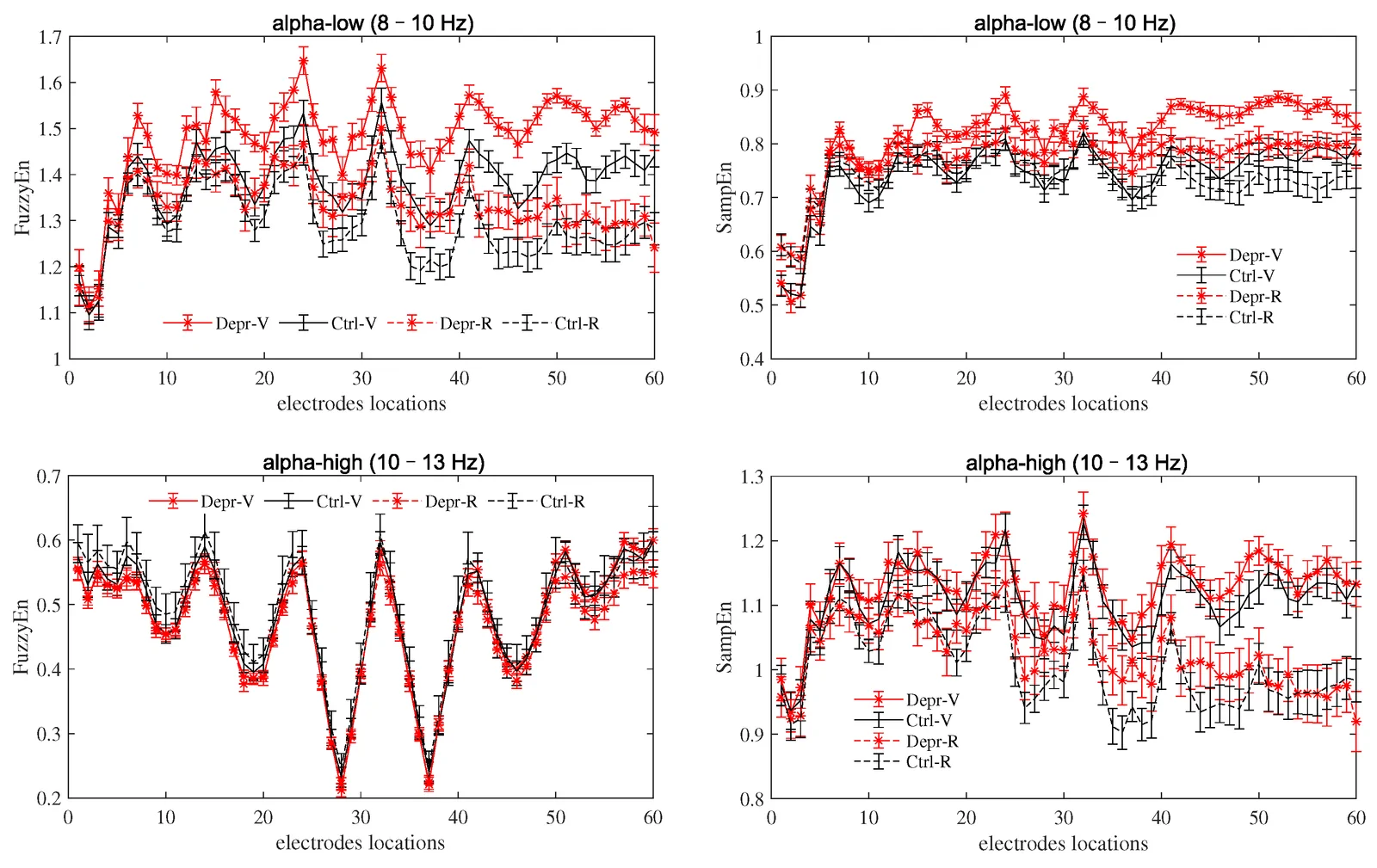
FuzzyEn and SampEn of depressed EEGs (mean ± standard error) in alpha-low (8–10 Hz) and alpha-high (10–13 Hz) bands. Electrode locations 1–60 in x-coordinate are same as those in Figure 5.

We further subdivided the alpha band into alpha-low (8–10 Hz) and alpha-high (10–13 Hz) bands to explore more refined neural activity patterns [39,40,41]. Within the alpha-low range, SampEn exhibited a superior discriminative effect in distinguishing depressive EEG from healthy controls more clearly, as is illustrated in Figure 6.

Thus, when depressed patients are exposed to visual stimuli, their brain generates more intense neural responses in the alpha-low range, as reflected by the higher dynamic complexity of EEG signals. Meanwhile, FuzzyEn could not identify the complex features of depressive EEG within the alpha-low range, suggesting its relative insensitivity to neural activity changes induced by visual stimulation in depressed individuals. Physiologically speaking, the alpha-low band EEG signals are predominantly localized to the parieto-occipital lobe, a brain region that functions as the primary hub for visual information processing [42]. The significantly increased dynamic complexity of alpha-low range EEGs in the left occipital lobe suggests that patients with depression display a pronounced stress response during visual stimulation paradigms. This observation offers substantial implications for elucidating the underlying mechanisms of visual processing deficits in the depressive population.

From the comparative analysis, both SampEn and FuzzyEn effectively characterize the abnormally high complexity of the occipital region of depressed EEGs. Theoretically, fuzzyEn more accurately measures the vector distances and probability distributions of the system but does not have a significant advantage in real-world signal analysis, and SampEn effectively quantifies the dynamic complexity of the frequency-band depressed EEGs, especially in the alpha band.

## 4. Discussions

FuzzyEn measures vector distances with higher precision via exponential fuzzy functions. Nevertheless, FuzzyEn has inferior performance to SampEn on model tests, and it also fails to outperform SampEn in depressive EEG signals, particularly for frequency band analysis. This phenomenon might be attributed to the fact that FuzzyEn retains detailed signal fluctuations [11,12,43] including noise and interference, rendering it more sensitive to disturbances. In contrast, SampEn screens the vector distance using the kernel function, and this typical coarse-grained method is advantageous in anti-noise performance. Sharing the same aspects, though fuzzy refinement can capture fine-grained signal details and boost measurement precision, fuzzy permutation, fDES and related fuzzy methods substantially elevate computational complexity [17,19,20,21,22] while making algorithms far more susceptible to noise and artifacts. Therefore, precise signal feature detection inherently comes at the cost of heightened vulnerability to interference. When the target signal exhibits limited noise contamination, FuzzyEn can generate more accurate estimations of dynamical complexity. For signals with unknown system characteristics or heavy noise pollution, however, SampEn represents a more robust alternative.

The association between higher dynamical complexity in the occipital lobe and pathological depression deserves further attention. Several resting-state studies have observed aberrant occipital nonlinear dynamics in major depressive disorder, predominantly manifested as increased signal irregularity within the alpha and beta frequency bands, stemming from impaired visual cortex arousal and sensory gating deficits [44]. Our complementary occipital findings extend the existing literature by revealing that depressive brain dysfunctions are not confined to frontocortical reward circuits but also involve the atypical nonlinear oscillation of the visual cortex during visual stimulus encoding [30]. Moreover, we attributed the significant intergroup differences in entropy metrics within the occipital lobe primarily to physiological visual processing functions. However, the reinforcement learning paradigm designed by Cavanagh et al. [23] integrates multiple cognitive components including reward and punishment evaluation, attentional allocation, motor output, and decision-making. Simply ascribing elevated occipital dynamical complexity to visual input processing fails to account for these confounding cognitive factors [31]. Thus, finding out whether differences in brain regions do occur in depressed EEGs in the resting state or conducting other types of experiments targeting different brain functions could be the next steps in research.

The current research has certain limitations requiring further discussion. Regarding the parameters for phase space reconstruction, we adopted a trial-and-error strategy that enumerates all parameter combinations within a predefined range and selects the optimal set based on output performance. Multiple systematic parameter selection approaches have been proposed in the existing literature, such as the mutual information method, the C-C algorithm [45,46], and Cao’s method [47]. However, our preliminary tests revealed that these methods yield highly inconsistent outcomes across different brain regions and individual subjects, while their underlying theoretical foundations differ substantially. For these reasons, we did not employ such automated algorithms in this work. Instead, we combined experimental observations with phase space theory while accounting for computational overhead, signal sampling frequency, and probability estimation to determine the optimal parameter configuration. Second, surrogate data theory was adopted to validate the chaotic characteristics of simulated time series, a standard technique widely utilized in nonlinear dynamical analysis. Nevertheless, our evaluation framework for noise robustness remains limited and requires further expansion. On one hand, only a narrow range of noise types was incorporated into the simulation experiments [43]. On the other hand, the binary threshold judgment rule ought to be substituted with rank-based p-values or z-scores for more rigorous statistical testing. These limitations lay a solid foundation and point out clear directions for our follow-up comprehensive investigations.

## 5. Conclusions

This study systematically compares the performance of SampEn and FuzzyEn in quantifying dynamic complexity through a logistic chaotic sequence model test and clinical analysis of depressed EEG data.

Surrogate data theory shows that SampEn achieves validated nonlinear feature detection under a lower SNR than FuzzyEn, thus showing superior anti-noise robustness.

Both SampEn and FuzzyEn exhibit insensitivity to parameter variations, and SampEn outperforms FuzzyEn in detecting depression-related complexity alterations in the alpha band (8–13 Hz), especially the low-alpha sub-band (8–10 Hz).

In summary, SampEn is more suitable for noisy signal analysis due to its coarse-grained vector distance selection strategy. It should be noted that the parameter selection and findings remain to be further validated on more representative datasets.

## Figures and Tables

**Table 1:** The FuzzyEn and SampEn of the logistic series and its surrogate data with AWGN.

SNR (dB)	1	2	3	4	5	6	7	8	9	10
FuzzyEn (*m*2)	1.034	0.990	0.950	0.909	0.874	0.854	0.821	0.795	**0.774**	**0.739**
F-2.5S	1.025	0.980	0.944	0.903	0.872	0.848	0.819	0.794	**0.775**	**0.753**
SampEn (*m*2)	1.960	1.974	1.961	1.966	**1.961**	**1.970**	**1.971**	**1.973**	**1.972**	**1.948**
S-2.5S	1.953	1.959	1.955	1.958	**1.963**	**1.971**	**1.973**	**1.974**	**1.979**	**1.987**
FuzzyEn (*m*3)	0.921	0.884	0.851	0.808	0.776	0.744	0.722	0.697	0.681	**0.652**
F-2.5S	0.915	0.8752	0.8413	0.803	0.770	0.743	0.722	0.694	0.677	**0.658**
SampEn (*m*3)	1.965	1.963	1.967	1.967	1.962	1.953	1.971	**1.967**	**1.968**	**1.952**
S-2.5S	1.944	1.948	1.953	1.948	1.951	1.957	1.967	**1.969**	**1.979**	**1.976**

Notes: F-2.5S and S-2.5S denote the 2.5th percentiles of the FuzzyEn and SampEn of the surrogate data; bold values indicate that the entropy values of the logistic series were lower than the 2.5th percentiles of the surrogate data.

## Data Availability

The data that support the findings of this study are openly available from on the PRED+CT website. https://predict.cs.unm.edu/, accession no. d003 and d006.
